# Immobilization of Fibronectin-Loaded Polyelectrolyte Nanoparticles on Cardiovascular Material Surface to Improve the Biocompatibility

**DOI:** 10.1155/2019/5478369

**Published:** 2019-10-31

**Authors:** Shihui Liu, Youdong Hu, Rongrong Tao, Qingwei Huo, Lin Wang, Chunzhi Tang, Changjiang Pan, Tao Gong, Nenggui Xu, Tao Liu

**Affiliations:** ^1^Medical College of Acu-Moxi and Rehabilitation, Guangzhou University of Chinese Medicine, Guangzhou 510006, China; ^2^Department of Geriatrics, The Affiliated Huai'an Hospital of Xuzhou Medical College, Huai'an 223002, China; ^3^Jiangsu Provincial Key Laboratory for Interventional Medical Devices, Huaiyin Institute of Technology, Huai'an 223003, China

## Abstract

Vascular stent interventional therapy is the main method for clinical treatment of coronary artery diseases. However, due to the insufficient biocompatibility of cardiovascular materials, the implantation of stents often leads to serious adverse cardiac events. Surface biofunctional modification to improve the biocompatibility of vascular stents has been the focus of current research. In this study, based on the structure and function of extracellular matrix on vascular injury healing, a novel fibronectin-loaded poly-l-lysine/heparin nanoparticles was constructed for stent surface modification. *In vitro* blood compatibility evaluation results showed that the nanoparticles-modified surface could effectively reduce platelet adhesion and activation. *In vitro* cellular compatibility evaluation results indicated that the nanocoating may provide adequate efficacy in promoting the adhesion and proliferation of endothelial cells and thereby accelerate endothelialization. This study provides a new approach for the surface biological function modification of vascular stents.

## 1. Introduction

Cardiovascular diseases (CVDs), such as coronary artery diseases (CADs), continue to be the leading cause of death globally [1]. Although the extensive clinical application of vascular stents has significantly improved the survival rate of patients with CADs, adverse cardiac effects caused by in-stent thrombosis and restenosis often lead to implantation failure [2]. With the in-depth exploration of the mechanism of pathological response induced by material implantation, it is widely accepted that the rapid endothelialization on the surface of cardiovascular materials after implantation is an ideal way to reduce the risk of postoperative complications. Currently, in-situ regulation of platelets and vascular cells behavior via surface biological function design and microenvironment construction has become a hot topic [3].

In the human body, the repair of vascular endothelium depends largely on the existence of vascular basement membrane [4], which is a kind of extracellular matrix composed of laminin, collagen, nestin, and heparin sulfate polysaccharide. Because of the important role of extracellular matrix components in cell growth, extracellular matrix proteins are also commonly used in surface modification of biomaterials to promote the growth behavior of vascular cells. Matrix proteins and some adhesive proteins, such as fibronectin (Fn) [5, 6], laminin (Ln) [7, 8], and collagen (Col) [9, 10], may bind to the specific receptors that are expressed on endothelial cells (ECs) and initiate a variety of intracellular pathways so as to promote a series of biological behaviors, such as cell adhesion, migration, growth, and proliferation. However, extracellular matrix components can not only promote cell growth but also lead to platelet adhesion and activation and then cause thrombosis [11, 12], which greatly limits the application of extracellular matrix coating in the field of cardiovascular materials. Therefore, it is necessary to find a way to construct the biological coating that may provide adequate properties to inhibit the function of protein molecules in promoting coagulation reaction without affecting the positive efficacy on endothelium regeneration.

Construction of multifunctional layer containing anticoagulant molecules and adhesive proteins on the surface of cardiovascular materials would be helpful to realize the selective regulation of the biological behavior of blood components and vascular cells and then to inhibit thrombus and restenosis, as well as accelerate endothelialization. However, in the current research on surface biological modification of cardiovascular materials, many functional coatings were often found having few beneficial effects on the direction of vascular neointimal regeneration. The main reasons are as follows: First, the limitations of the construction methods of biological coatings on materials surface, for example, the introduction of biomolecules on the surface of materials by covalent immobilization, may lead to the decrease of biological activity [13], while the biological coatings constructed by the interaction between biomolecules (such as electrostatic assembly) also have the defects of intermolecular functional domain shielding and lack of stability. Second, the constructed biological functional layer is not effective for a long time in vivo, so it is easy to release or degrade in the complex dynamic environment of the body and lose its functional activity rapidly. In recent years, multifunctional nanoparticles have gradually shown great potential in the controlled release of biomolecules and the regulation of cellular behavior [14, 15]. Compared with other controlled release systems, the unique nanoeffect of nanoparticles system greatly increases its loading capacity and can separate the active molecules from the surrounding environment to avoid rapid inactivation [16].

In our previous studies, we constructed a novel heparin/poly-l-lysine nanoparticle for surface modification of cardiovascular materials and found that the nanocoating can effectively improve the anticoagulant properties [17]. In order to further enhance its potential to promote endothelial regeneration, fibronectin was loaded onto nanoparticles to provide sufficient cell binding sites to accelerate endothelialization after stent implantation. It was found that the nanoparticles could be successfully immobilized to the dopamine-coated surface and the modified surface exhibits proper functions of inhibiting coagulation reaction and promoting the adhesion and proliferation of endothelial cells. This study provides a feasible method for surface biological function modification of cardiovascular materials.

## 2. Materials and Methods

### 2.1. Materials and Reagents

316L stainless steel (316L SS) was processed into round shape (Φ10 mm, ∼1.2 mm thickness) and mirror-polished. Fibronectin (Fn), dopamine (DA), and poly-l-lysine (PLL, MW 150∼300 KDa) were purchased from Sigma-Aldrich. Low molecular weight heparin sodium (Hep, ≥180 U/mg), acid orange II (AO II), toluidine blue O (TBO), and rhodamine 123 were purchased from Aladdin BioChem Technology Co., Ltd (Shanghai, China). 0.0067 M phosphate-buffered saline (PBS, pH 7.4) was used for PLL, Hep, and Fn solution preparation.

### 2.2. Preparation of Fn-Loaded Hep/PLL Nanocoating on 316L SS Surface

**Figure 1:**
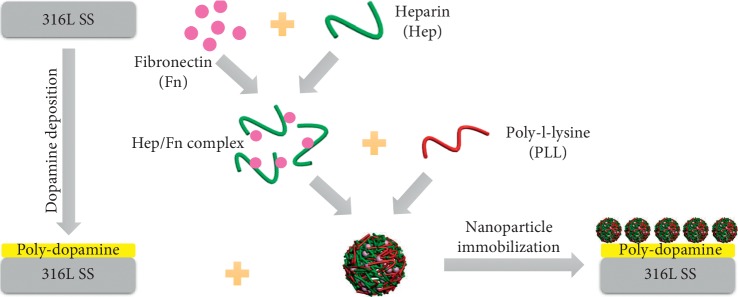
Schematic drawing of fibronectin-loaded nanoparticles fabrication and immobilization.

As shown in Figure 1, the nanoparticles were immobilized on the DA-coated 316L SS surface. A detailed protocol for DA coating deposition can be referred to our previous study [7]. For nanoparticle preparation, 100 *μ*g/ml Fn solution was mixed with an equal volume of 10 mg/ml heparin solution and incubated at 37°C for 3 h. Then, 0.5 mg/ml PLL solution was added dropwise to equal volume of Hep/Ln mixture under high-frequency ultrasonic conditions to formulate the Fn-loaded Hep/PLL nanoparticles. After that, DA-coated 316L SS was placed in a 24-well plate and 0.5 ml of nanoparticle suspension was added to the sample surface. The plate was subsequently placed in an air bath shaking table and incubated at 37°C for 12 h with gentle shaking (60–65 rpm) (termed as SS-DA-NPF). The sample modified with only Hep/PLL nanoparticle was set as the control group and termed as SS-DA-NP.

### 2.3. Size and Zeta Potential Analysis

The size, zeta potential, and particle dispersion index (PDI) of prepared nanoparticles were characterized by dynamic light scattering using a ZETA-SIZER, MALVERN Nano-2S90 (Malvern Ltd., Malvern, UK).

### 2.4. FTIR Assay

The alteration of surface chemical structure during surface modification was detected by Fourier transform infrared spectroscopy (FTIR). The FTIR assay is carried out by using the attenuated total reflection (ATR) model on the Tensor-27 infrared spectrum (Bruker, Germany). The infrared adsorption between 4000 and 650 cm^−1^ was recorded at room temperature and atmospheric conditions.

### 2.5. Quantitative Characterization of Amine Group and Heparin Exposing Density

Surface amine density was detected by the AO II method. The samples were initially immersed in 1 ml of 0.5 mM AO II solution (in pH = 3 HCl) and incubated at 37°C for 6 hours with gentle shaking. Afterward, the samples were thoroughly rinsed with HCl solution (pH = 3). Then, the samples were placed on a filter paper, and the surface was dried with a gentle blow. Next, the samples were immersed in 1 ml of NaOH water solution (pH = 12) and shaken at 37°C for 30 minutes. Finally, 150 *μ*l of NaOH solution mixed with eluted AO II was transferred into a 96-well plate, and the absorbance value was detected at 485 nm. The AO II molar concentration was parallel to that of the amine group.

TBO assay was prepared to detect heparin exposing density of nanoparticle-modified surfaces. First, the samples were placed in a centrifuge tube, and 5 ml of 0.04 wt.% TBO solution (in 0.01 M HCl/0.2 wt.% NaCl solution) was added. The tube was subsequently placed in an air bath shaker and incubated at 37°C for 4 hours with gentle shaking (∼60 rpm). Next, the samples were thoroughly rinsed with UP water and immersed in 5 ml of 80% ethanol/0.1 M NaOH (v/v = 4/1) solution. After being shaken for 10 minutes, the solution mixed with eluted TBO was transferred to a 96-well plate, and the absorbance value was detected at 530 nm.

### 2.6. Water Contact Angle and AFM Assay

The alteration of surface hydrophilicity during nanoparticles modification was detected by using a DSA25 contact goniometer (Krüss GmbH, Germany). The test was carried out at room temperature, and at least 3 independent points of each sample were measured.

The morphology of nanoparticles-modified surface was detected by atom force microscopy (AFM) (Bruker Innova, Germany). The test was conducted at room temperature, and the image was processed by NanoScope Analysis software.

### 2.7. *In Vitro* Platelet Adhesion Assay

Human whole blood was taken from healthy volunteers. The blood that was anticoagulated by using 3.8% sodium citrate (1/10 in volume ratio) was firstly centrifuged at 1500 rpm for 15 min to acquire platelet-rich plasma (PRP). The sample was then placed in a 24-well plate, and 0.5 ml PRP was added to each well to ensure that the sample was fully immersed in PRP. Subsequently, the plate is placed in a constant-temperature water bath shaker and incubated at 37°C for 2 hours with gentle shaking. After that, the sample was rinsed with normal saline and the platelets adhered on the surface were fixed in 2.5% glutaraldehyde for over 12 hours. For platelets fluorescence staining, 50 *μ*l of 1 *μ*g/ml rhodamine 123 solution was added to each sample surface and incubated at room temperature for 20 min. The morphology of adherent platelets was observed under an inverted fluorescence microscope.

P-selectin expression was detected to evaluate the platelet activation profile on different sample surface. A detailed protocol can be referred to our previous study [7].

### 2.8. ECs Adhesion and Proliferation Assay

Endothelial cells (ECs) were isolated from human umbilical cord veins and cultured in DMEM/F12 medium containing 15% fetal bovine serum (FBS) and 20 *μ*g/ml ECGS. The culture medium was changed every 2 days. The cells were isolated with 0.25% trypsin/EDTA solution when the fusion degree was over 85% and adjusted to 1 × 10^5^ cell/ml by using fresh culture medium for subsequent cell seeding.

316L SS and SS-DA that were used for cell seeding were presterilized by high pressure steam, and the immobilization of nanoparticles was carried out under aseptic conditions. All samples were placed in a 24-well plate, and 1 ml EC suspension was added to each well and incubated at 37°C under 5% CO_2_ for 1 and 3 days, respectively. At each time point, the culture medium was removed and the fresh medium containing 10% CCK-8 reagent was added to the culture for 3.5 hours. After that, the absorbance of the culture medium at 450 nm was detected to evaluate the cell proliferation activity on different sample surfaces. Then, the samples were fixed in 2.5% glutaraldehyde solution for subsequent cell fluorescence staining. A detailed experimental protocol can be referred to our previous study [7].

### 2.9. Statistical Analysis

At least three independent experiments were performed for the tests described above. The data were analyzed with the software SPSS 22.0. Statistical evaluation of the data was performed using one-way ANOVA. The probability value *p* < 0.05 was considered significant.

## 3. Results and Discussion

### 3.1. Size and Zeta Potential Analysis

According to Table 1, the average size of blank nanoparticles is 313 ± 19 nm. After loading Fn, the particle size decreased slightly (287 ± 26 nm), which was mainly due to the increase of the internal force caused by the interaction between Fn and heparin, which enhanced the compactness of the particles. The results of zeta potential show that the incorporation of Fn has no significant effect on the charge of nanoparticles, which may be partly related to the lower Fn concentration when compared with heparin and PLL. On the other hand, the absolute values of zeta potential of both NP and NPF are higher than 20 mV, which indicates that the particle system has adequate stability. Besides, for nanoparticle system, it is generally considered that the particle size is more uniform when the dispersion coefficient (PDI) is less than 0.2, and the lower the dispersion coefficient, the better the uniformity. The results show that the PDI values of the prepared nanoparticles are lower than 0.2, indicating that both NP and NPF are of favorable uniformity.

### 3.2. FTIR Assay

**Figure 2:**
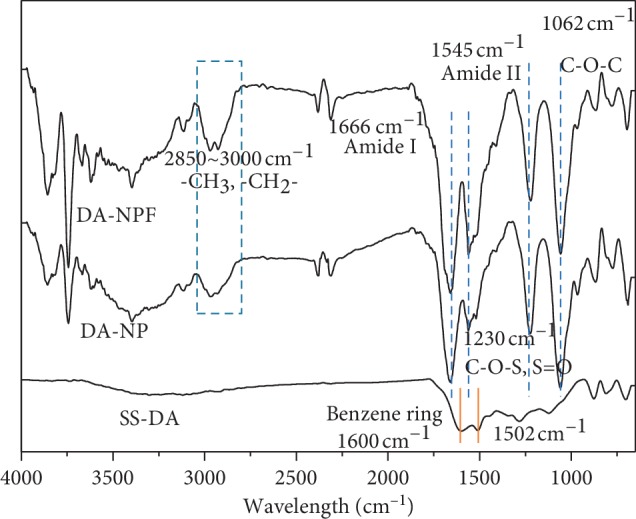
Surface chemical composition determined by FTIR spectra.

Figure 2 shows the infrared spectroscopy of NP- and NPF-modified surface. According to the result, for the DA-coated surface, the peaks related to benzene ring structure could be observed at 1600 cm^−1^ and 1502 cm^−1^, which indicates the success deposition of dopamine coating. By comparison, obvious new absorption peaks were emerged after immobilization of NP and NPF. In detail, the absorption peaks in the range of 2850–3000 cm^−1^ are related to the vibration of -CH_3_ and -CH_2_- groups. The new peaks at 1666 cm^−1^ and 1545 cm^−1^ are related to the stretching vibration of amide I and amide II from PLL molecular structure, respectively. In addition, the new peak at 1230 cm^−1^ is related to the vibration of C-O-S and S=O groups, and the peak at 1062 cm^−1^ is related to the vibration of C-O-C group. Both of these two peaks further demonstrated the existence of heparin.

The above results show that both NP and NPF are successfully immobilized on the dopamine-coated surface. On the other side, when compared with the NP-modified surface, the peak intensity of amide bond on the NPF-modified surface is obviously increased at the same coordinate, which may be related to the existence of Fn in nanoparticles, or to the difference in the immobilization density between NP and NPF.

### 3.3. Quantitative Characterization of Heparin and Amine Exposing Density

**Figure 3:**
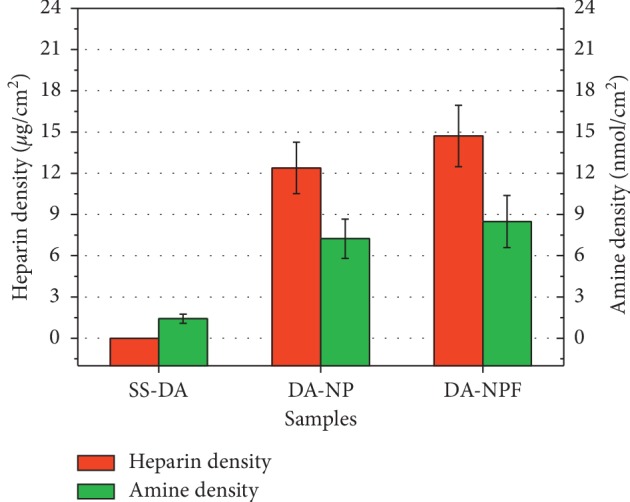
Quantitative characterization result of amine group and heparin exposing density of different sample surfaces (mean ± SD, *n* = 6).

The quantitative results of amine group and heparin exposing density on the surface of different samples are shown in Figure 3. The results show that there is a small amount of amine group (1.42 ± 0.33 nmol/cm^2^) on the DA surface; however, after NP immobilization, the exposure density of amine group was greatly increased (7.2 ± 1.4 nmol/cm^2^). This was mainly due to the PLL used in this study is an amino-rich polycationic electrolyte. When compared with NP, the amine exposing density on NPF-modified surface is further increased, which was mainly related to the existence of the Fn molecule. The immobilization of nanoparticles on the DA-coated surface largely depends on the chemical reaction between amino groups and quinoid groups; thereby, the increase of amino density may promote the immobilization of nanoparticles on the surface. As demonstrated, the exposure density of heparin on the NP surface was 12.38 ± 1.87 *μ*g/cm^2^, while that of the NPF-modified sample increased to 14.72 ± 2.23 *μ*g/cm^2^. The results also proved the conclusions of the FTIR results.

On the other hand, according to our previous study [17], it is found that a certain amine group (0–13 nmol/cm^2^) and heparin exposing density (3–10 *μ*g/cm^2^) on material surface are helpful to the selective regulation of blood and cellular behavior. In this study, the surface amine and heparin exposing densities detected are in the above range, so the modified surface may provide favorable efficacy in regulating the biological behavior of platelet and vascular cells.

### 3.4. Surface Physical and Chemical Properties

**Figure 4:**
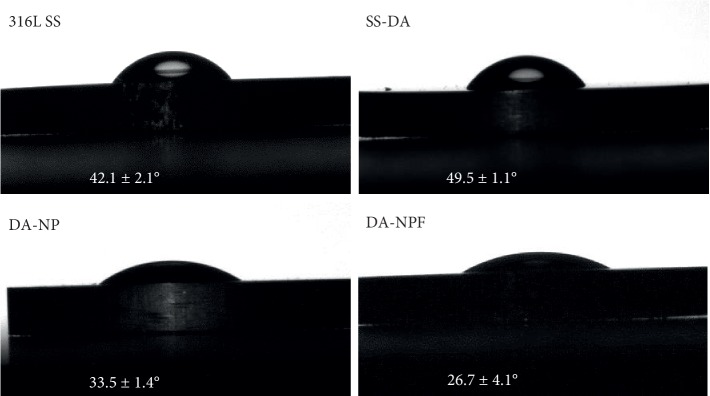
Alteration of surface hydrophilicity during surface modification (mean ± SD, *n* = 6).

The hydrophilic and hydrophobic properties of the material surface can be simply characterized by the measurement of water contact angle. According to Figure 4, when compared with 316L SS, the water contact angle increased slightly after dopamine deposition. This is mainly due to the hydrophobic benzene ring structure on the SS-DA surface. After immobilization of NP, the water contact angle was significantly decreased (^*∗*^*p* < 0.05); this is mainly due to that the heparin and PLL molecules contained a variety of hydrophilic groups, such as-COOH, -NH_2_, and-SO_3_. In comparison, the hydrophilicity of NPF-modified surface was further increased. This may be related to the increase of heparin and amine exposing density on the surface, which increases the content of hydrophilic groups on the surface.

According to the study, when the water contact angle is in the range of 30∼60 degree, the biomaterial surface may facilitate cell adhesion and proliferation via preferentially adsorption of adhesive serum proteins, such as collagen, fibronectin, or vitronectin [18, 19]. In addition, the increase of hydrophilicity on the surface of the sample will trigger the desorption of viscous proteins [20], which is helpful to reduce the adhesion and aggregation of platelets on the surface of the material. In this study, thereby, the NP- and NPF-modified surface may provide adequate hydrophilicity to regulate platelets and vascular cells behavior.

**Figure 5:**
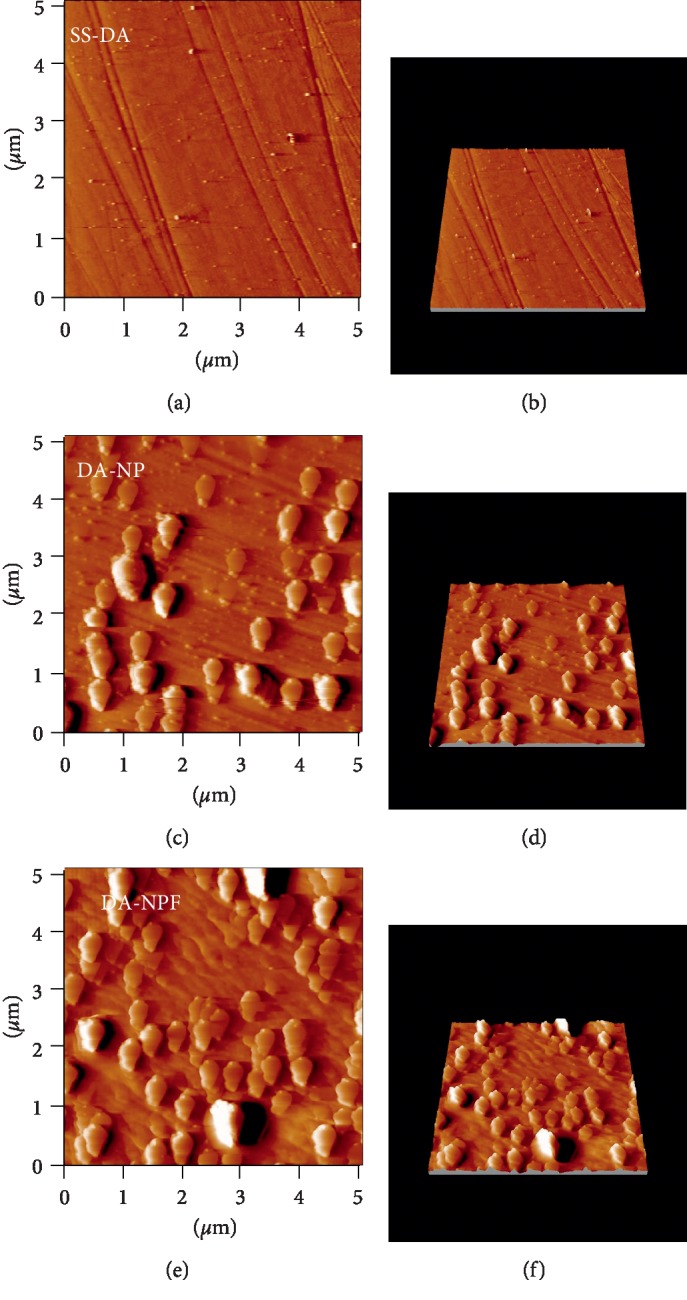
Morphology of nanoparticles-modified surface determined by AFM.

Figure 5 shows the AFM morphology of the sample surface before and after modification of nanoparticles. It can be observed that the SS-DA surface is relatively smooth with existence of few small particles, which is mainly formed during the self-polymerization of dopamine. In comparison, both NP- and NPF-modified surface showed obvious particle structure, and the particle size is about 300 to 400 nm, which is consistent with the results shown in Table 1. In addition, no obvious particle aggregation was found on the NP- and NPF- modified surface. This may be related to the high zeta potential value of prepared nanoparticles, which can effectively inhibit the agglomeration of particles during the process of immobilization.

### 3.5. *In Vitro* Platelet Adhesion Assay

**Figure 6:**
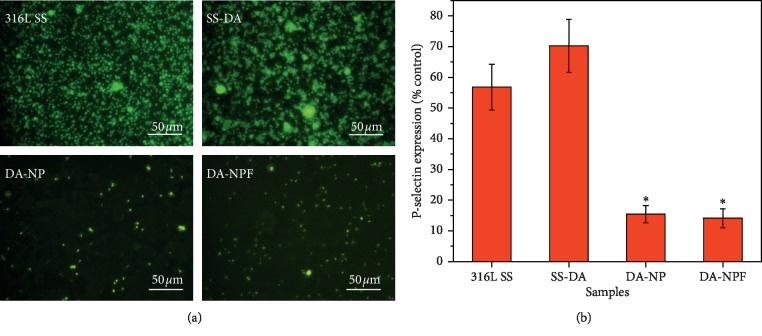
*In vitro* blood compatibility evaluation result. (a) Rhodamine 123 fluorescence staining of adhered platelets. (b) P-selectin semiquantitative characterization result (mean ± SD, *n* = 6; ^*∗*^*p* < 0.05 indicates significant difference in comparison with 316L SS and SS-DA).

As a blood contact device, favorable hemocompatibility was usually taken into first consideration. In this study, *in vitro* platelet adhesion and activation were prepared to evaluate the anticoagulation efficacy of the modified surface.  Figure 6(a) shows the results of rhodamine 123 staining of adherent platelets after incubation in platelet-rich plasma for 2 hours. It can be seen that there are a large number of platelets adhered to the surface of SS and SS-DA; in particular, serious aggregation of platelets could be observed on SS-DA surface, which indicates that the blood compatibility of the sample is poor. For the NP- and NPF-modified surfaces, the platelet adhesion density was dramatically decreased, and no obvious platelet aggregation and activation were found. P-selectin expression result further demonstrated that the NP- and NPF-modified surface may inhibit platelet activation (Figure 6(b)). Although numerous studies have shown that fibronectin is a procoagulant [21, 22], the biological function of the modified surface is closely associated with the synergistic effect of different biomolecules. The heparin density on the surface of NPF-modified samples is relatively high, which can effectively block the coagulation pathway, inhibit platelet adhesion and activation, and thereby improve blood compatibility.

### 3.6. ECs Adhesion and Proliferation Assay

**Figure 7:**
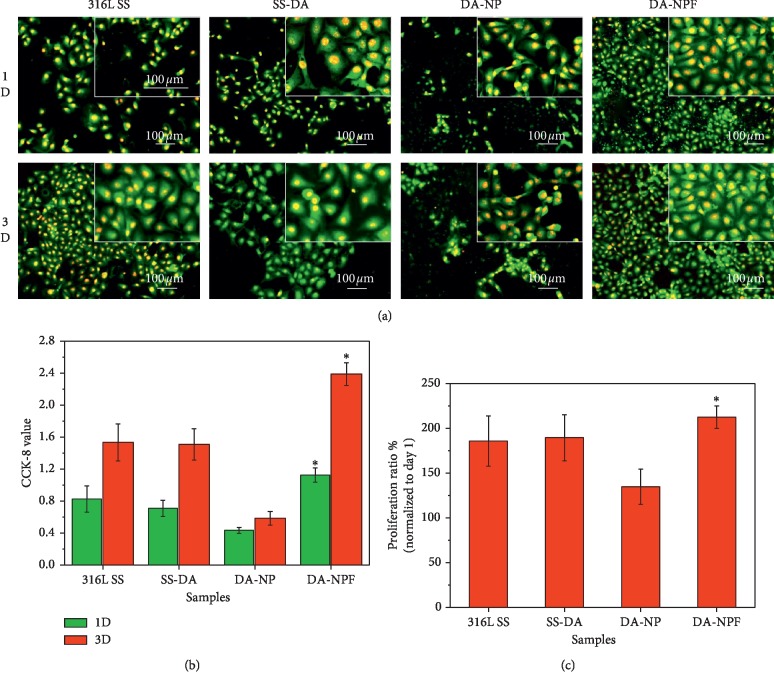
(a) Rhodamine 123 and DAPI fluorescence staining of adhered ECs after culture of 1 and 3 days. (b) Cell proliferation activity detected by CCK-8. (c) Cell proliferation ratio from day 1 to 3. (mean ± SD, *n* = 12; ^*∗*^*p* < 0.05 indicates significant difference).

The effect of modified surface on ECs adhesion and proliferation behavior is shown in Figure 7. Rhodamine 123 fluorescence staining result (Figure 7(a)) shows that ECs displayed a well-spread morphology on 316L SS and SS-DA surface. In comparison, the cell coverage area on NP-modified surface was significantly decreased and showed a shrinkage morphology after incubation for 1 and 3 days, which indicated the activity of adhered ECs was poor. For the NPF-modified surface, the ECs adhesion density was obviously increased, and the adherent cells showed classical cobblestone morphology. CCK-8 assay was used to further detect the proliferation activity of adherent cells. As shown in Figure 7(b), the NP-modified surface showed the lowest CCK-8 value after 1- and 3-day culture, while the proliferation activity was significantly increased (^*∗*^*p* < 0.05) on the NPF surface. The EC proliferation ratio from day 1 to 3 further demonstrates that surface modification with NPF may accelerate endothelialization (Figure 7(c)).

Rapid endothelialization is one of the most important requirements for the surface modification of vascular stents. For biofunctionalized surface, the biocompatibility is closely related to the surface physicochemical characteristics and biological properties. In particular, the synergistic effect of a variety of biomolecules plays an important role in the regulation of tissue-material interface response. In this study, the cell adhesion density and proliferation activity of NP-modified surface significantly decreased; this is mainly due to the high density of heparin on the modified surface. According to our previous study, the heparinized surface may inhibit cell growth when heparin-binding density is more than 10 *μ*g/cm^2^ [17]. This is mainly related to the high negative charge of heparin, which may trigger the blocking effect to some important intracellular signaling pathways that closely associate with cell adhesion and proliferation behavior [23–25]. On the other hand, although the NPF-modified surface showed higher heparin exposing density, the incorporation of Fn may bring abundant cell adhesion sites, and therefore, the cell proliferation activity was enhanced. In summary, the surface of NPF modified samples not only showed adequate efficacy of inhibiting platelet adhesion and activation but also exhibited favorable property of accelerating endothelialization, both of which are helpful to improve the clinical performance of biomaterials implanted into blood vessels.

## 4. Conclusion

In this study, novel Fn-loaded heparin/poly-l-lysine nanoparticles were constructed and immobilized to dopamine-coated 316L SS surface to improve the biocompatibility. It is found that the incorporation of Fn can improve the binding density of nanoparticles, which may contribute to enhancing the anticoagulant properties of the surface and thereby prevent the coagulation reaction caused by the presence of Fn. In addition, the Fn-loaded nanocoating was found to effectively improve the adhesion and proliferation of vascular endothelial cells on the material surface and thereby accelerate endothelium regeneration. Molecular biological mechanism of the synergistic effects among different biomolecules in vascular remodeling and endothelium regeneration will be investigated in future studies.

## Figures and Tables

**Table 1:** Size and zeta potential of prepared nanoparticles.

	Zeta potential (mV)	Size (nm)	PDI
NP	−27.3 ± 0.5	313 ± 19	0.102 ± 0.052
NPF	−26.2 ± 1.4	287 ± 26	0.049 ± 0.011

## Data Availability

All data and models used to support the findings of this study are available from the corresponding author upon request.
